# Unexpected giant negative area compressibility in palladium diselenide

**DOI:** 10.1093/nsr/nwad016

**Published:** 2023-01-20

**Authors:** Xingxing Jiang, Shengzi Zhang, Dequan Jiang, Yonggang Wang, Maxim S Molokeev, Naizheng Wang, Youquan Liu, Xingyu Zhang, Zheshuai Lin

**Affiliations:** New Functional Crystals Group, Key Laboratory of Functional Crystals and Laser Technology, Technical Institute of Physics and Chemistry, Chinese Academy of Sciences, Beijing 100190, China; Center of Materials Science and Optoelectronics Engineering, University of Chinese Academy of Sciences, Beijing 100049, China; New Functional Crystals Group, Key Laboratory of Functional Crystals and Laser Technology, Technical Institute of Physics and Chemistry, Chinese Academy of Sciences, Beijing 100190, China; University of Chinese Academy of Sciences, Beijing 100049, China; Center for High Pressure Science & Technology Advanced Research, Beijing 100094, China; School of Materials Science and Engineering, Peking University, Beijing 100871, China; Laboratory of Crystal Physics, Kirensky Institute of Physics, SB RAS, Krasnoyarsk 660036, Russia; Department of Physics, Far Eastern State Transport University, Khabarovsk 680021, Russia; International Research Center of Spectroscopy and Quantum Chemistry, Siberian Federal University, Krasnoyarsk 660041, Russia; New Functional Crystals Group, Key Laboratory of Functional Crystals and Laser Technology, Technical Institute of Physics and Chemistry, Chinese Academy of Sciences, Beijing 100190, China; University of Chinese Academy of Sciences, Beijing 100049, China; New Functional Crystals Group, Key Laboratory of Functional Crystals and Laser Technology, Technical Institute of Physics and Chemistry, Chinese Academy of Sciences, Beijing 100190, China; University of Chinese Academy of Sciences, Beijing 100049, China; New Functional Crystals Group, Key Laboratory of Functional Crystals and Laser Technology, Technical Institute of Physics and Chemistry, Chinese Academy of Sciences, Beijing 100190, China; University of Chinese Academy of Sciences, Beijing 100049, China; New Functional Crystals Group, Key Laboratory of Functional Crystals and Laser Technology, Technical Institute of Physics and Chemistry, Chinese Academy of Sciences, Beijing 100190, China; Center of Materials Science and Optoelectronics Engineering, University of Chinese Academy of Sciences, Beijing 100049, China; University of Chinese Academy of Sciences, Beijing 100049, China

**Keywords:** negative area compressibility, Lifshitz mechanism, charge transfer

## Abstract

Negative area compressibility (NAC) is a counterintuitive ‘squeeze–expand’ behavior in solids that is very rare but attractive due to possible pressure–response applications and coupling with rich physicochemical properties. Herein, NAC behavior is reported in palladium diselenide with a large magnitude and wide pressure range. We discover that, apart from the rigid flattening of layers that has been generally recognized, the unexpected giant NAC effect in PdSe_2_ largely comes from anomalous elongation of intralayer chemical bonds. Both structural variations are driven by intralayer-to-interlayer charge transfer with enhanced interlayer interactions under pressure. Our work updates the mechanical understanding of this anomaly and establishes a new guideline to explore novel compression-induced properties.

## Teaser

Two-dimensional ‘squeeze–expand’ property is detected in a layered van der Waals crystal of palladium diselenide and its unexpected giant magnitude ascribes to the intra- and interlayer charge transfer under pressure.

## INTRODUCTION

As a counterintuitive departure from normal squeeze–shrink response behavior, negative compressibility is an intriguing mechanical phenomenon in which the size of a substance is anomalously elongated along specific direction(s) under hydrostatic pressure [1–3]. The negative compressibility effect is highly attractive not only for fundamental compression-induced mechanisms [2,4] but also for pressure–response applications in materials [5]. From the viewpoint of compression–expansion dimensions, negative compressibility can occur either along one direction (negative linear compressibility, NLC) or along two orthogonal directions (negative area compressibility, NAC), while that along three orthogonal directions (negative volume compressibility) is forbidden for solids in thermodynamically closed systems [6,7]. Clearly, a higher dimension of negative compressibility can provide more freedom to modulate the compression-induced properties and enhance the performances in materials, and thus exploration of the NAC effect is the core topic in this work. In fact, the NAC effect can provide a unique approach to subtly manipulate fundamental physical properties and to revolutionarily improve the performance in materials, with examples including modification of the atomic-stacking anisotropy to creation of superconductivity under pressure [8] and production of piezoelectric pressure sensors in which the sensitivity is enhanced by at least one order of magnitude [1,9,10]. However, compared with the rarely occurred NLC behavior, the NAC effect is even less discovered; to date, only 10 NAC materials have been found [8–17].

To date, there are three mechanisms known to occur in NAC materials, in which the Lifshitz mechanism is most established and dominantly governs NAC materials, including TlGaSe_2_ [16], NaV_2_O_5_ [17], KBe_2_BO_3_F_2_ (*R*32 and *R*-3*c* phases) [13,14], silver(I) tricyanomethanide [10] and Zn(CH_3_COO)_2_·2H_2_O [11]. In this mechanism, NAC behavior can be generated by rigid flattening of ripples in corrugated 2D-like layers due to extrusion of neighboring counterparts under pressure [18]. The other two NAC mechanisms include twisting and phase-transition mechanisms, which result from twisting of microscopic units in a flexible 3D framework or from the rearrangement of atomic stacking near a pressure-induced phase-transition point, as exemplified by oxalic acid dehydrate [12], [Zn(L)_2_(OH)_2_]_n_.guest [9], 2MeBzIm[15] and CrAs [8]. Note that the Lifshitz mechanism is based on a (quasi)rigid unit model in which the flattening of ripples is quite limited, resulting in a small NAC magnitude despite a wide NAC pressure range. In contrast, the NAC magnitudes arising from twisting and phase-transition mechanisms are generally large, but their NAC pressure ranges are often small because these types of NAC behaviors occur only in the narrow pressure ranges before framework collapse or around the phase-transition point. The pressure range of the NAC directly determines the pressure in which the NAC is applicable and a wide pressure range signifies a high pressure tolerance of the NAC property. As the NAC magnitude and pressure range are two fundamental parameters that directly determine the capability to regulate compression-induced properties and enhance performance in NAC materials, the search for new NAC materials with both large magnitude and wide pressure range and the elucidation of the intrinsic mechanism are highly desirable.

In this work, we focus on 2D van der Waals (vdW) materials and note that their anisotropic structures largely match Lifshitz's structural feature. Moreover, the interatomic interaction across the layers in 2D vdW materials can be significantly modified by exerted pressure, e.g. in black phosphorus [19], molybdenum disulfide [20] and FePS_3_ [21], which is favorable to the generation of novel mechanical properties. By the screening criterion that the cations and anions in the neighboring layers need to be directly contacted with each other under pressure, PdSe_2_ attracted our great attention. It has been shown that a pressure-induced transition from a low-spin to a high-spin electronic configuration on Pd^2+^ cations leads to superconductivity in PdSe_2_ [22], indicating that the intralayer and interlayer interaction can be significantly modified under pressure. A high-pressure phase-transition study qualitatively suggested that the *a*- and *b*-axes in PdSe_2_ are elongated under pressure [23]. By utilizing high-pressure X-ray diffraction, herein, we demonstrate that PdSe_2_ not only has an NAC pressure range from 3.94 to 11.37 GPa with the largest NAC pressure range in all known NAC materials but also has an NAC amplitude of –13.14(240)/TPa, which exceeds the maximum value in all Lifshitz-governed NAC materials. The first-principles calculations, combined with high-pressure Raman measurements, reveal that the unexpected giant NAC behavior in PdSe_2_ originates from ripple flattening in the conventional Lifshitz mechanism as well as anomalous elongation of intralayer chemical bonds in 2D layers. More fundamentally, both structural variations are driven by intralayer-to-interlayer charge transfer under pressure.

## RESULTS

**Figure 1.**
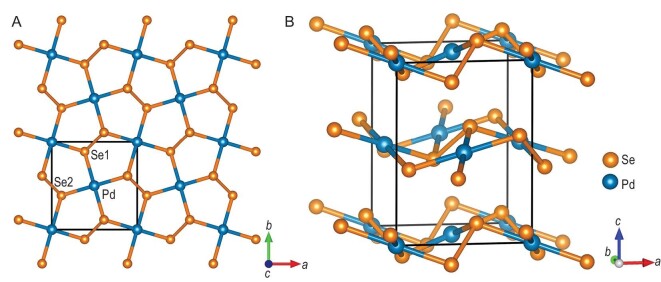
Crystal structure of PdSe_2_ viewed along (A) the *c*-axis and (B) the *b*-axis. The Se atoms at different symmetric positions are indicated by Se1 and Se2.

Under ambient pressure, PdSe_2_ crystalizes in the orthorhombic P*bca* space group (low-pressure phase, LP phase) with cell parameters of *a* = 5.7377(7) Å, *b* = 5.8603(7) Å and *c* = 7.6846(11) Å [24]. The palladium atoms are 4-fold coordinated with selenium atoms to form planar [PdSe_4_] quadrilaterals, which are further connected with one another by sharing corner selenium atoms to construct infinite [PdSe_2_]_∞_ layers (Fig. 1a). The [PdSe_2_]_∞_ layers are corrugated along the (*a, b*) plane and the ripples are formed by triangular pyramids with Se atoms as vertices (Fig. 1b). The [PdSe_2_]_∞_ layers are stacked along the *c*-axis, giving rise to highly anisotropic atomic-stacking features. Note that the electrically neutral [PdSe_2_]_∞_ layers are mutually adhered by weak vdW interactions and that the palladium and selenium atoms in the adjacent layer are aligned along the *c*-axis. The layer-to-layer approach under pressure leads these atoms to strongly interact with each other.

**Figure 2.**
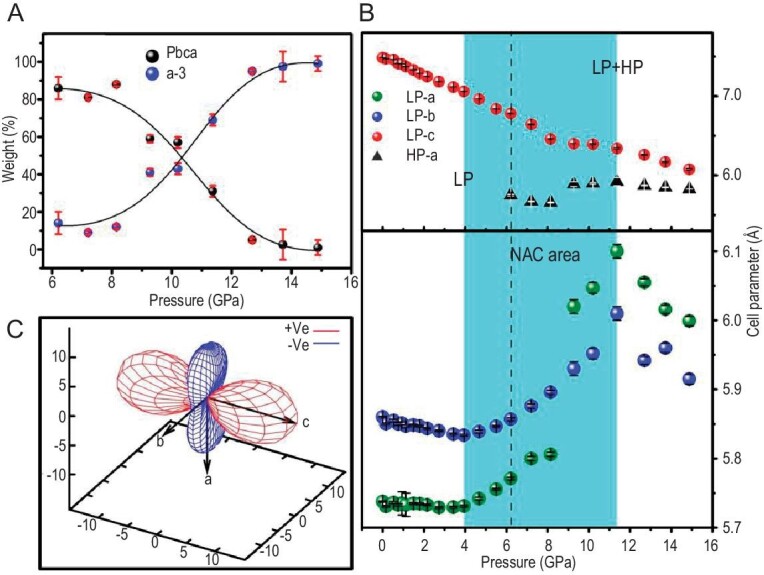
Compressive behavior of PdSe_2_. (A) The weight of the LP and HP phase versus pressure. (B) Refined cell parameters versus pressure. The NAC pressure range for the LP phase is highlighted in cyan. (C) Spatial distribution of linear compressibility for the LP phase plotted by using the PASCal program [26].

**Figure 3.**
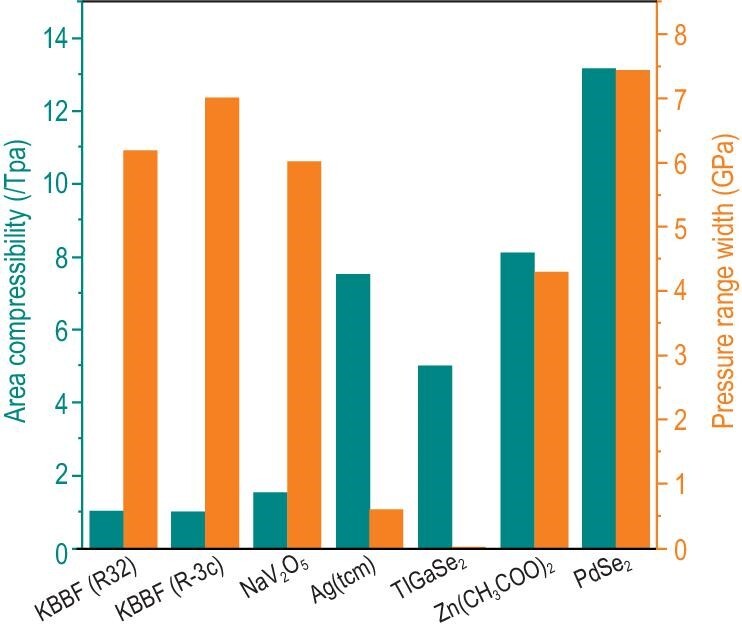
Comparison between the NAC magnitude and pressure range in PdSe_2_ and all other known Lifshitz-governed NAC materials.

High-pressure X-ray diffraction patterns of PdSe_2_ from ∼0 to 14.88 GPa are plotted in Supplementary Fig. S1. The structural refinement (Supplementary Fig. S2) reveals that a pressure-induced phase transition occurs at 6.21 GPa from the LP phase to a high-pressure (HP) phase crystallized in the cubic space group P*a-3* (with a pyrite-type structure) and that the phase transition is totally reversible (Supplementary Figs S3–S5). The HP-phase structure is consistent with that determined by Soulard *et al.* [23] and ElGhazali *et al.* [22]. Interestingly, the LP phase remains over the whole measured pressure range, although its weight ratio decreases with increasing pressure (Fig. 2a). Moreover, the LP-phase structure alone exhibits an anomalous cell-parameter evolution versus pressure (see Fig. 2b and Supplementary Table S1). (i) The (*a, b*) plane area remains almost constant at <3.94 GPa and decreases by 0.1% for the *a*-axis and 0.5% for the *b*-axis, with fitted compressibility coefficients of 0.23(12)/TPa and 0.85(13)/TPa, respectively. These compressibility values are comparable to that in diamond, the stiffest material in nature, and can be categorized as a typical zero-area compressibility [25]. (ii) As the pressure increases from 3.94 to 11.37 GPa, the *a*- and *b*-axes are anomalously elongated by 6.4% and 3.0%, respectively, corresponding to linear compressibility coefficients of –8.90(215)/TPa and –4.24(25)/TPa. Thus, the (*a, b*) plane exhibits NAC behavior with an area compressibility of –13.14(240)/TPa (Fig. 2c). This NAC magnitude is the largest among all known Lifshitz-governed NAC materials and is comparable to materials exhibiting the twisting and phase-transition NAC mechanisms, along with the widest pressure range of 7.43 GPa (from 3.94 to 11.37 GPa); see Fig. 3 and Supplementary Table S2. (iii) The NAC behavior terminates at >11.37 GPa, and both the *a*- and *b*-axes are normally contracted as pressure increases (i.e. exhibiting positive compressibility). In the whole measured pressure range (0–14.88 GPa), the *c*-axis monotonously decreases with a normal (positive) compressibility.

**Figure 4.**
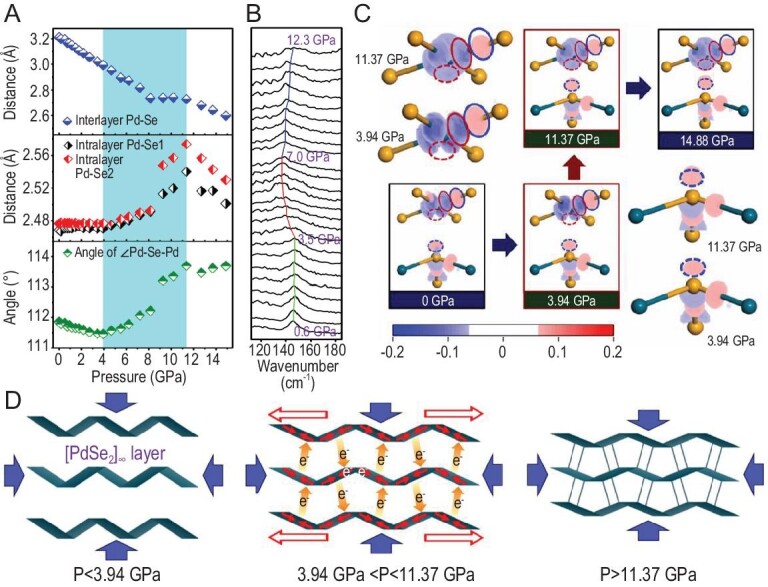
Mechanism of the NAC behavior in PdSe_2._ (A) Bond length and angle evolution versus pressure. The NAC pressure range is highlighted in cyan. (B) Shifting of the Raman peak characterizing intralayer Pd–Se bonds versus pressure. Insensitivity, softening and hardening of the Raman modes are highlighted by green, red and blue curves, respectively. (C) Charge-difference density contours at various pressures. Palladium and selenium are represented by black blue and orange balls, respectively. The blue and red solid-line ellipses indicate the charge-gain and charge-loss areas along the intralayer Pd–Se bonds, while the dashed-line ellipses indicate the charge-gain and charge-loss areas along the interlayer Pd–Se pair. The numbers in the color scale represent the gain and loss electron densities (unit:/Å^3^). The darker the red (or blue) color, the more electrons gained (or lost) in the area are. The size and shape for each ellipse are set to be kept constant with respect to pressure in order to make the comparison of charge-difference density explicit. The magnified charge-difference densities on the [PdSe_2_] layer and on the neighboring Se atom in the NAC pressure range are displayed on the left and right corners, respectively. (D) Schematic for the mechanical mechanism in PdSe_2_. When the hydrostatic pressure *P* < 3.94 GPa, the modification of the interlayer charge transfer is weak, suggesting that the interlayer interaction is also weak and that zero-area compressibility behavior occurs in the (*a *and* b*) plane. As 3.94 GPa < *P* < 11.37 GPa, the change in charge transfer becomes prominent, which makes the ripples flatten and the Pd–Se bonds elongate synergistically in the [PdSe_2_]_∞_ layers, resulting in NAC behavior. When the charge transfer finishes (*P* > 11.37 GPa), NAC behavior terminates and positive compressibility is obtained. The whole PdSe_4_ coordination units are simplified to the quadrilateral and Pd–Se bonds generated by the interlayer charge transfer are represented by vertical thin rods.

PdSe_2_ manifests unexpected giant NAC behavior over both magnitude and pressure ranges, exceeding all known Lifshitz's structural motifs. To elucidate the intrinsic mechanism, we investigated the pressure-induced variations in the atomic positions in PdSe_2_. However, the X-ray diffraction (XRD) patterns generally deteriorate under high pressure, which further interferes with the coexistence of LP and HP phases in PdSe_2_ and severely hinders accurate determination of pressure-induced variation in atomic positions. To overcome this problem, high-precision first-principles simulations were carried out to calculate the pressure-dependent atomic positions in PdSe_2_ (Fig. 4a and Supplementary Table S3). Accordingly, the following characteristics for the variation of atomic geometry can be observed. (i) Over the pressure range from 0 to 3.94 GPa, the size of the PdSe_2_ layers along the (*a, b*) plane is unchanged, with the intralayer Pd–Se bond lengths remaining almost constant (Fig. 4a), despite the adjacent layers prominently approaching. This means that since the interlayer vdW interaction is negligibly small, the applied stress is dominantly released between the adjacent layers. Thus, the intralayer atomic positions are only minimally influenced by pressure and zero-area compressibility behavior occurs. (ii) Over the pressure range from 3.94 to 11.37 GPa, the adjacent layers significantly interact with one another, which strongly squeezes the [PdSe_2_]_∞_ layers. This effect increases the intralayer Pd–Se–Pd angles from 111.5° to 113.7° (Fig. 4a), i.e. the ripples within the layers are flattened. Moreover, the intralayer Pd–Se bonds are anomalously elongated (from 2.471 to 2.540 Å and from 2.477 to 2.574 Å for Pd–Se1 and Pd–Se2 bonds, respectively; see Fig. 4a), implying that the enhanced interlayer interaction has a strong influence on the intralayer configuration. The increase in Pd–Se–Pd angles and the elongation of intralayer Pd–Se bonds synergistically generate the large NAC behavior in the (*a, b*) plane. Remarkably, the contribution from the elongation of intralayer Pd–Se bonds to NAC is much larger (∼72%) than that from the flattening of ripples (∼28%), revealing the significant role of bond elongation in enhancing the NAC effect. (iii) Under pressures of >11.37 GPa, both intralayer Pd–Se–Pd angles and Pd–Se bonds in the [PdSe_2_]_∞_ layer are decreased as pressure increases, corresponding to positive compressibility.

Clearly, the atomic structure evolution of NAC in PdSe_2_ cannot be fully ascribed to the Lifshitz mechanism. That mechanism is established on a (quasi)rigid rod-like structure model, which considers only the flattening of ripples and not the NAC contribution from changes in chemical bond lengths. Under normal circumstances, atoms approach each other under pressure to contract chemical bonds, so the elongation of the intralayer Pd–Se bonds in PdSe_2_ is quite abnormal. To experimentally confirm this reasoning, we performed HP Raman measurements (Supplementary Fig. S6). As shown in Fig. 4b, the position of Raman peak @ ∼140 cm^−1^, which characterizes the stretching of intralayer Pd–Se bonds (displayed in Supplementary Fig. S7b) [22], tends to remain constant, soften and then harden as the pressure increases from 0.6 to 12.3 GPa, corresponding to the invariance, elongation and contraction of these bond lengths, respectively. The HP Raman measurements are consistent with the atomic simulations and confirm the anomalous elongation of intralayer Pd–Se bonds over the NAC pressure range. Moreover, since the Se–Se bonds in the HP phase are weaker than those in the low-pressure phase, the corresponding characteristic Raman peak at a lower frequency of ∼200 cm^−1^ appears and becomes gradually prominent with the pressure application in the former phase compared with that at 260 cm^−1^ in the latter phase (Supplementary Fig. S6). After returning from high pressure, the Raman spectrum is reverted to the initial one (Supplementary Fig. S6) and no hysteresis is built.

To further investigate the mechanism behind the increase in Pd–Se–Pd angles and the anomalous elongation of intralayer Pd–Se bonds, first-principles charge-difference density maps versus pressure in PdSe_2_ are plotted in Fig. 4c, from which several characteristics can be deduced. (i) At ambient pressure (at ∼0 GPa), the charge-loss densities on Pd exhibit typical characteristics of ${d}_{{x}^2 - {y}^2}$ orbitals, suggesting that these orbitals are emptied as intralayer Pd–Se bonds are formed. Thus, according to Hund's rule, the lowest-energy electronic configuration of the square-planar coordinated Pd^2+^ (*d*^8^) cation is the single-state ^1^*E*_1_ state, i.e. ${d}_{{{( {{z}^2} )}}^2}{d}_{{{({x}^2 - {y}^2)}}^0}$ [27]. As the pressure increases from ∼0 to 3.94 GPa (below the initial NAC pressure), although the [PdSe_2_]_∞_ layers significantly approach each other under pressure, the charge-difference densities in the highlighted ellipse areas are almost unchanged. This indicates that no charge transfer occurs between the intralayer Pd–Se bonds and between the [PdSe_2_]_∞_ layers, verifying that the weak interlayer interaction character remains almost unchanged. (ii) From 3.94 to 11.37 GPa, along the intralayer charge-gain areas (the blue solid-line ellipses in Fig. 4c) gradually decrease, as do those in the charge-loss areas (the red solid-line ellipses in Fig. 4c). Clearly, the electrons are transferred back from Se to Pd in the (*a, b*) plane in the NAC pressure range. This charge transfer decreases the bond order (or strength) of the intralayer Pd–Se bonds and in turn elongates their bond lengths. Meanwhile, along the interlayer Pd–Se bonds, the densities in both charge-loss (the red dashed ellipses in Fig. 4c) and charge-gain (the blue dashed ellipses in Fig. 4c) areas are prominently increased. This suggests that quite a few charges are transferred from Pd to Se across the layers to compensate for the charge transfer along the intralayer Pd–Se bonds to keep the valence state on palladium as normal as possible. Accompanying the interlayer charge-transfer process, the interlayer Pd–Se interaction is gradually enhanced, which squeezes the ripples in the [PdSe_2_]_∞_ layers. Moreover, the charge-loss areas on Pd with the shape of ${d}_{{x}^2 - {y}^2}$ orbitals gradually vanish, while those with the shape of ${d}_{{z}^2}$ are gradually enhanced. Thus, the electronic states on Pd change from ${d}_{{{( {{z}^2} )}}^2}{d}_{{{({x}^2 - {y}^2)}}^0}$ to the intermediate ${d}_{{{( {{z}^2} )}}^{2 - \delta }}{d}_{{{({x}^2 - {y}^2)}}^\delta }$ during the NAC process. (iii) Above 11.37 GPa (exceeding the NAC pressure range), the electron densities in the charge-gain and charge-loss areas remain almost unchanged as the pressure increases. The NAC structural modifications are terminated and the normal positive compressibility is exhibited along all three axes.

As mentioned above, since the rigid flattening of ripples under pressure is generally weak, the magnitude of the NAC effect owing to the normal Lifshitz mechanism is usually quite small (see Supplementary Table S2). Our study on PdSe_2_ reveals that, accompanying the increase in interlayer interaction between the [PdSe_2_]_∞_ layers in the NAC pressure range, the charges are spatially redistributed from the intralayer to the interlayer regions, which flattens the ripples and weakens (and then elongates) the intralayer Pd–Se bonds. The significantly enhanced NAC behavior in PdSe_2_ is attributed to an additional effect from the elongation of intralayer Pd–Se bonds induced by intralayer-to-interlayer charge transfer. The NAC mechanism in PdSe_2_ is schematically displayed in Fig. 4d. It should be emphasized that the NAC mechanism in PdSe_2_ is not a type of phase-transition mechanism, as this anomalous compression–expansion response occurs only in the single phase (LP phase) from 3.94 to 11.37 GPa, despite the emergence of the HP phase at 6.1 GPa. Because the number of XRD peaks is much greater than that of the refined cell parameters, phase transition and a abrupt change in the lattice parameter between 8.15 and 9.27 GPa induced by the phase transition would not interfere the determination of the NAC in the LP phase (see the discussion in Supplementary Section S1).

It is known that negative compressibility can be coupled with novel physical and chemical properties in materials, including superconductivity [8], prominent hydrogen bond bending [2], ultralow bulk modulus [3,28] and long-life phosphorescence [5]. Moreover, negative compressibility materials are of great significance to the fabrication and application of state-of-the-art devices under high pressure, such as high-performance sonar sensors [4], signal converters in deep-sea cables [29] and even shock absorbers in body armor [3,6]. Therefore, the unexpected giant NAC effect in PdSe_2_ has significant implications for the understanding of its compression-induced physicochemical properties and for further combination with the tunable energy band gap, strong optical anisotropy and high electron field-effect mobility in PdSe_2_ [30–32] to promote its uses in advanced systems under pressure. For example, the charge-transfer process in PdSe_2_ prominently influences the electrical transporting property [22] and the NAC provides a new approach to finely tune the performance in field emission devices, for example, by pressure [33].

## DISCUSSION

In summary, as first found in 2D vdW materials, we demonstrate a rarely occurring NAC behavior in PdSe_2_ with an increase in both NAC amplitude and pressure range by >50% over all other Lifshitz-governed NAC materials. We discovered that, apart from the flattening of ripples as considered by the Lifshitz mechanism, the anomalous elongation of intralayer chemical bonds contributes to ∼70% of the NAC effect, which is the main reason for the significantly enhanced NAC effect in PdSe_2_. Both structural variations are driven by intralayer-to-interlayer charge transfer with enhanced interlayer interactions under pressure. Our research paves a new path for the design and exploration of NAC materials with large magnitudes and wide pressure ranges. Namely, in materials that tend to generate charge transfer with enhanced 2D interlayer interactions under pressure, the intralayer chemical bonds can be effectively elongated to improve the NAC effect. More fundamentally, owing to highly anisotropic structures and unique electronic features, 2D vdW materials manifest intriguing electronic, optical, thermal and magnetic properties [34–40]. The charge-transfer mechanism presented in this work establishes an intrinsic relationship between the structural modification and electron behavior in 2D vdW materials under pressure. The discovery of NAC materials with large magnitudes and wide pressure ranges is helpful to provide an ideal platform to understand and modulate novel compression-induced properties, such as superconductivity, piezoposphorescence and other quantum effects.

## METHODS

### Sample preparation

The sample was purchased from Nanjing MKNANO Tech. Co., Ltd (www.mukenano.com) and was directly used after purchase without further purification.

### HP X-ray diffraction

HP X-ray diffraction patterns were collected at the 4W2 beam line of the Beijing Synchrotron Radiation Facility (BSRF). The X-ray beam at a wavelength of 0.6199 Å was focused into a 36 × 12 μm^2^ spot using a Kirkpatrick–Baez mirror. Well-ground sample powder was placed in a hole with a diameter of 200 μm in a pre-indented stainless-steel gasket with a thickness of 40 μm (Supplementary Fig. S10). Systematic diamond anvil cell (DAC) with a culet diameter of 400 μm exerted pressure, and a mixture of methanol and ethanol at a ratio of 4 : 1 was adopted to act as the pressure-transmitting medium to generate hydrostatic pressure. DAC creates high pressure by trapping a sample between the cutlet faces of two diamonds (as depicted in Supplementary Fig. S11). A ruby with a diameter of ∼5 μm was placed in a sample cavity and pressure was calibrated by measuring the fluorescence shift of the ruby [41]. The Debye-Sherrer powder method was employed to collect the diffraction patterns with the X-ray transmitting through the diamond and samples, and the diffraction patterns were recorded by using a Pilatus image plate. Using FIT2D software, 2D diagrams were integrated into 1D patterns. The crystal structures were refined by using the Rietveld method [42] using TOPAS 4.2 software. The compressibility was fitted by using the PASCal program [26]. The method and formula to calculate the compressibility are as follows.

The key calculations in PASCal involve the determination of orthogonal strains. The starting point is the transformation from crystallographic axes **A***_i_* to orthogonal axes **E***_i_*. The orientation of the **E**_i_ axes is arbitrary: in this work we made use of the Institute of Radio Engineers convention where **E**_3_ is parallel to the *c* crystallographic axis, **E**_1_ is parallel to a* and **E**_2_ = **E**_3_×**E**_1_. The corresponding change-of-basis transformation is described by the square matrix **M**:


\begin{eqnarray*} {\rm{{\boldsymbol E} = {\boldsymbol M} \times \ {\boldsymbol A}}} \end{eqnarray*}

The strain ϵ, corresponding the lattice change between the initial and final pressures, was given as the symmetric part of the product of **M** at the initial pressure with its inverse at the final pressure, i.e. by defining:


\begin{eqnarray*} {{\bf e}}\ {\rm{ = \ }}{{\bf M}}_{{\rm{final}}}^{{\rm{-1}}}{\rm{\ \times }}\ {{{\bf M}}}_{{\rm{initial}}}\ {\rm{-\ }}{{\bf I}} \end{eqnarray*}

**M_final_** and **M_initial_** are the transformation matrix between the crystallographic and orthogonal coordinates after and before strain, respectively.

We obtained:


\begin{eqnarray*} {{\bf \varepsilon }}\ {\rm{ = \ }}\frac{{\rm{1}}}{{\rm{2}}}\left( {{{\bf e}}{\rm{ \,\,+\,\, }}{{{\bf e}}}^{\rm{T}}} \right) \end{eqnarray*}

The eigenvalues ϵ_i_ and eigenvectors **x**_i_ of the matrix **ϵ** are the principal strains and the principal axes, respectively. The derivatives of these principal strains with respect to pressure give the coefficients of linear compressibilities ${\alpha }_i's$ along the principal axes:


\begin{eqnarray*} {\rm{\ }}{\alpha }_i = \ - {\left( {\frac{{\partial {\varepsilon }_i}}{{\partial p}}} \right)}_T \end{eqnarray*}

Then the area compressibility *α*_(_*_i, j_*_)_ of the (*i, j*) plane is:


\begin{eqnarray*} {\alpha }_{\left( {i,j} \right)} = {\alpha }_i + {\alpha }_j \end{eqnarray*}

### HP Raman spectrum

Pressure was exerted and calibrated using the same method with X-ray diffraction. Raman spectra were collected from 50 to 800 cm^−1^ using a Via-Reflex equipped with a solid-state laser with a wavelength of 532 nm. To improve the signal-to-noise ratio of the spectra, 10 integrations were carried out on polycrystalline samples with an integration time of 10 s at a nominal resolution of 1 cm^−1^ and a precision of 1 cm^−1^.

### First-principles simulation

First-principles calculations were performed by using plane-wave pseudopotential density functional theory (DFT) [43], implemented in the CASTEP package [44]. The generalized gradient approximation with the Perdew, Burke and Ernzerhof (PBE) [45] functionals was adopted to describe the exchange and correlation (XC) energy. The effective ion–electron interactions were modeled by using norm-conserving pseudopotentials [46], in which Pd 4*d*^10^ and Se 4*s*^2^4*p*^4^ electrons were treated as valence electrons. Plane-wave energy cut-off of 600 eV and Monkhorst–Pack [47] *k*-point spanning <0.04/Å in the Brillouin zone were chosen. The long-range vdW interactions were modeled by using Tkatchenko and Scheffler's scheme [48]. Raman spectrum was calculated by using the linear response mechanism [49].

## Supplementary Material

nwad016_Supplemental_File
