# Supplementary material for: Unexpected giant negative area compressibility in palladium diselenide
Source: Natl Sci Rev. 2023 Jan 20;10(9):nwad016. doi: 10.1093/nsr/nwad016 (PMC10411663; doi:10.1093/nsr/nwad016)
Supplement: nwad016_Supplemental_File [file nwad016_supplemental_file.docx]

**Supplemental Material for**

**Unexpected Giant Negative Area Compressibility in Palladium Diselenide**

Xingxing Jiang, ^†,1,2^ Shengzi Zhang, ^†,1,6^ Dequan Jiang,^3^ Yonggang Wang,^4^ Maxim S. Molokeev,^5,7,8^ Naizheng Wang,^1,6^ Youquan Liu,^1,6^ Xingyu Zhang,^1,6^ Zheshuai Lin*^1,2,6^

**Corresponding Author**

*E-mail: [zslin@mail.ipc.ac.cn](mailto:zslin@mail.ipc.ac.cn) (Z. L.)

^1^ New Functional Crystals Group, Key Laboratory of Functional Crystals and Laser Technology, Technical Institute of Physics and Chemistry, Chinese Academy of Sciences, Beijing 100190, China.

^2^Center of Materials Science and Optoelectronics Engineering, University of Chinese Academy of Sciences, Beijing100049, P.R. China.

^3^Center for High Pressure Science & Technology Advanced Research, Beijing 100094, China.

^4^School of Materials Science and Engineering, Peking University, Beijing 100871, China.

^5^Laboratory of Crystal Physics, Kirensky Institute of Physics, SB RAS, Krasnoyarsk 660036, Russia.

^6^University of Chinese Academy of Sciences, Beijing 100049, China.

^7^Department of Physics, Far Eastern State Transport University, Khabarovsk 680021, Russia.

^8^Siberian Federal University, Krasnoyarsk 660041, Russia.

^†^contributed equally to this work.

**Contents**

**Figure S1** XRD patterns of PdSe_2_ at various hydrostatic pressures between 0 and 14.88 GPa.

**Figure S2** Refinement plots of PdSe_2_.

**Table S1** Refined cell parameters and weight of the two phases at various hydrostatic pressures.

**Figure S3** XRD patterns of PdSe_2_ at various hydrostatic pressures between 0 and 12.19 GPa with pressure-loading and –unloading process.

**Figure S4** Refinement plots of PdSe_2_ with pressure-loading and –unloading process.

**Figure S5** Dependence of lattice volume on pressure.

**Table S2** Area compressibility (AC) and NAC pressure range of all known NAC materials.

**Table S3.** Bond length and angles evolution *versus* pressure.

**Figure S6** High-pressure Raman spectra of PdSe_2_ from 0.6 GPa to 12.3 GPa.

**Figure S7** Raman spectrum at ambient pressure and the atomic vibration assignment of the Raman peaks.

**Figure S8** Charge difference density contours of PdSe_2_ at various hydrostatic pressures from 0 to 14.88 GPa.

**Figure S9** Charge difference density contours of PdSe_2_ at some specified pressures.

**Figure S10** Photos of PdSe_2_ sample.

**Figure S11** Schematic for the setup of high pressure and X-ray.

**Figure S1** XRD patterns of PdSe_2_ at various hydrostatic pressures between 0 and 14.88 GPa.
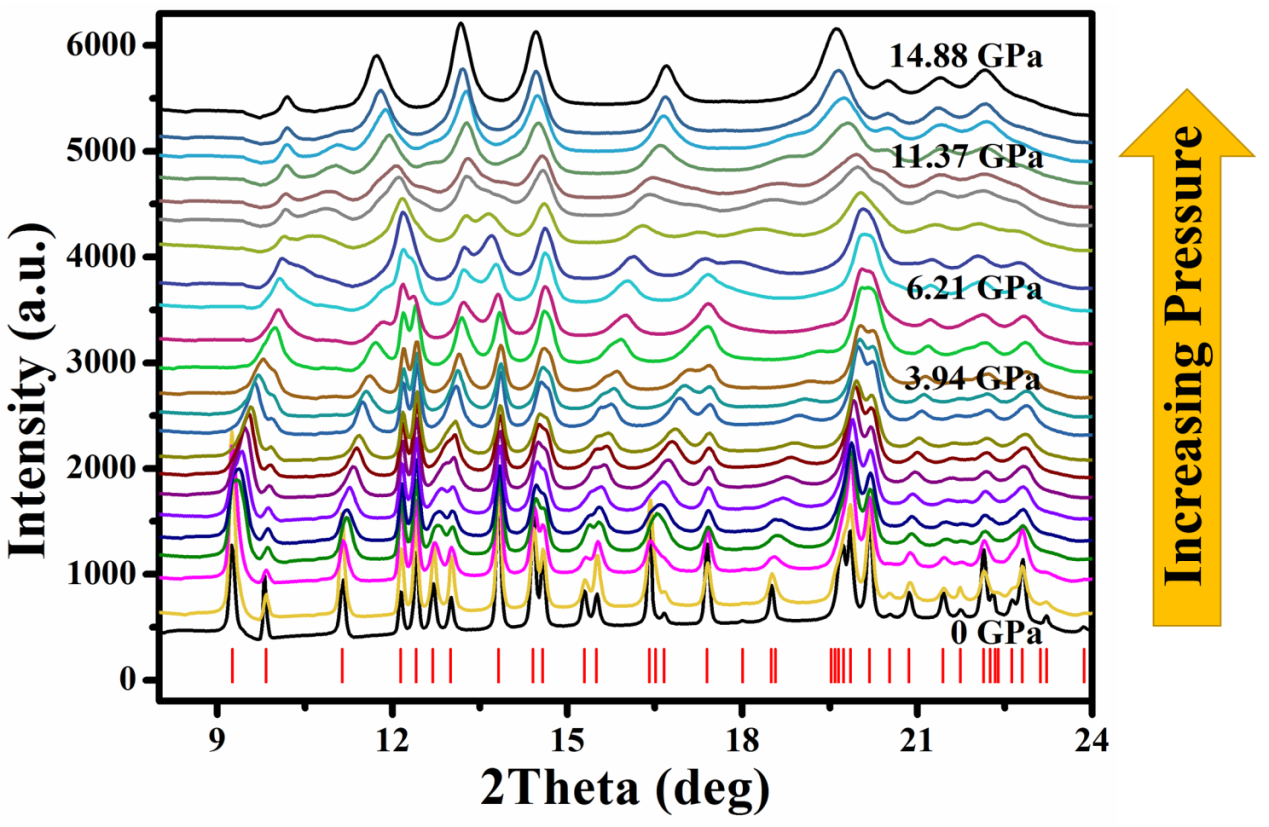


**Figure S2** Refinement plots of PdSe_2_ at: (a) 0 GPa, (b) 0.156 GPa, (c) 0.533 GPa, (d) 0.751 GPa, (e) 0.930 GPa, (f) 1.129 GPa, (g) 1.488 GPa, (h) 1.798 GPa, (i) 2.159 GPa, (j) 2.727 GPa, (k) 3.425 GPa, (l) 3.944 GPa, (m) 4.672 GPa, (n) 5.506 GPa, (o) 6.211 GPa, (p) 7.207 GPa, (q) 8.150 GPa, (r) 9.268 GPa, (s) 10.199 GPa, (t) 11.365 GPa, (u 12.693 GPa, (v) 13.713 GPa, (w) 14.875 GPa.**
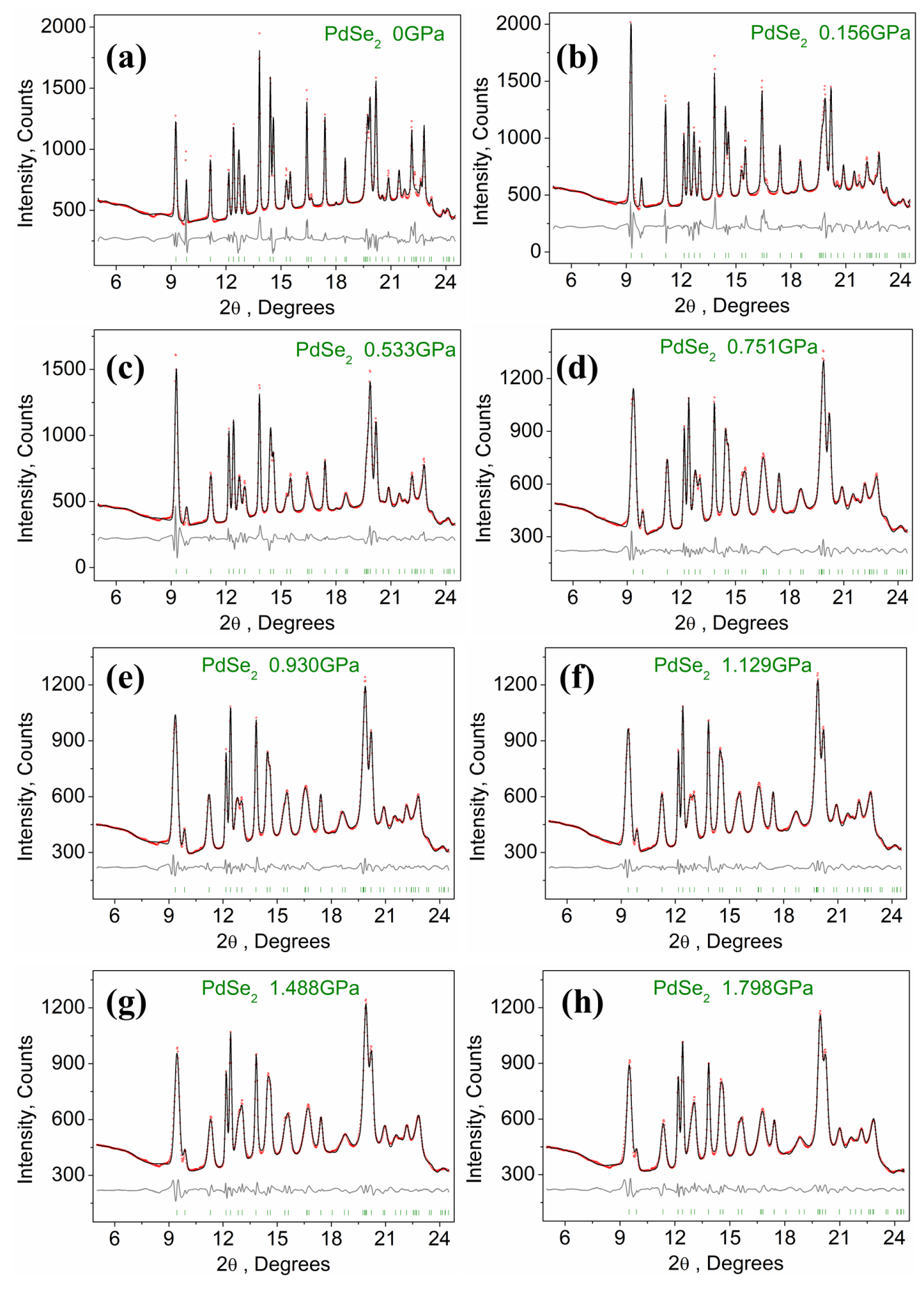

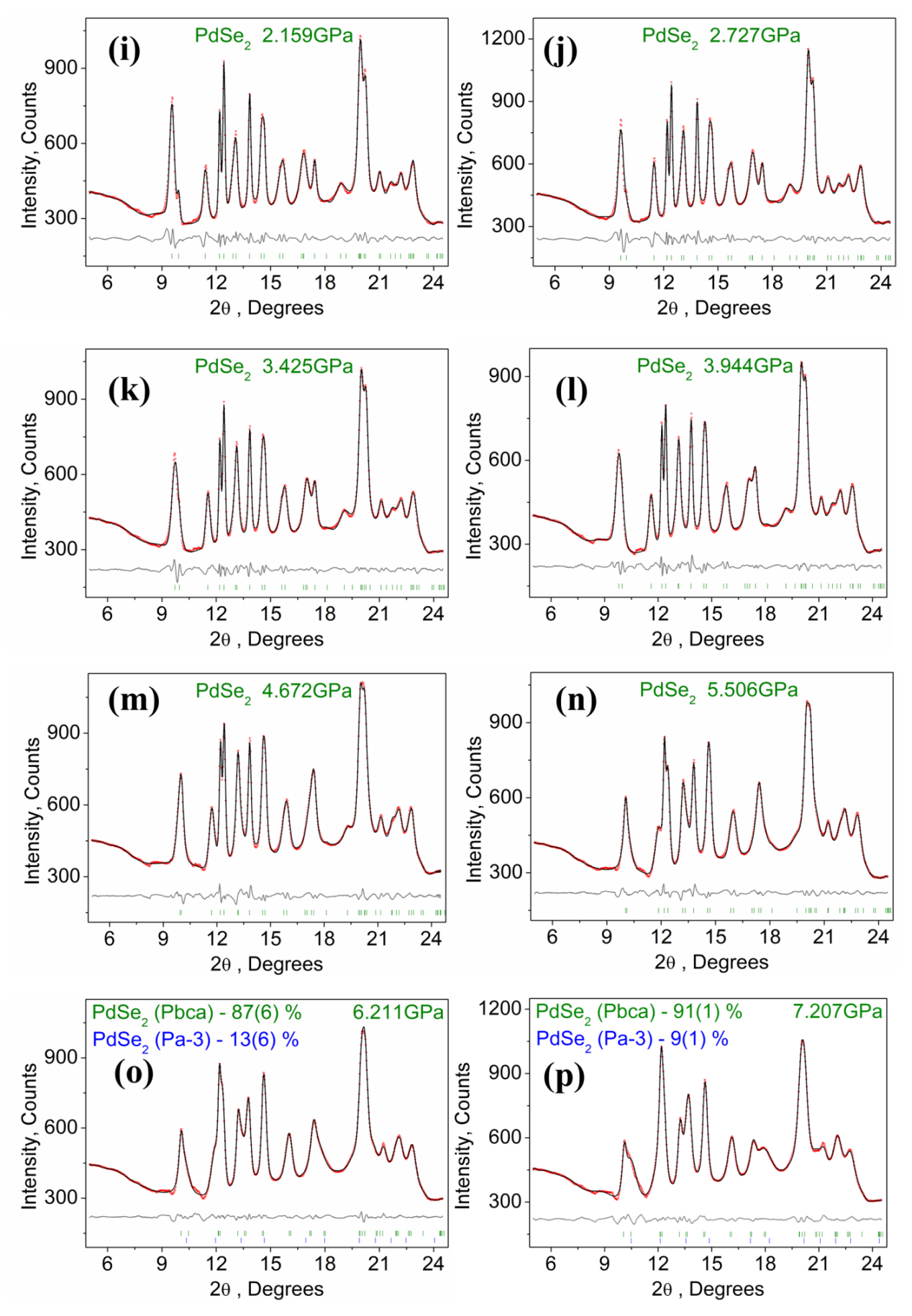

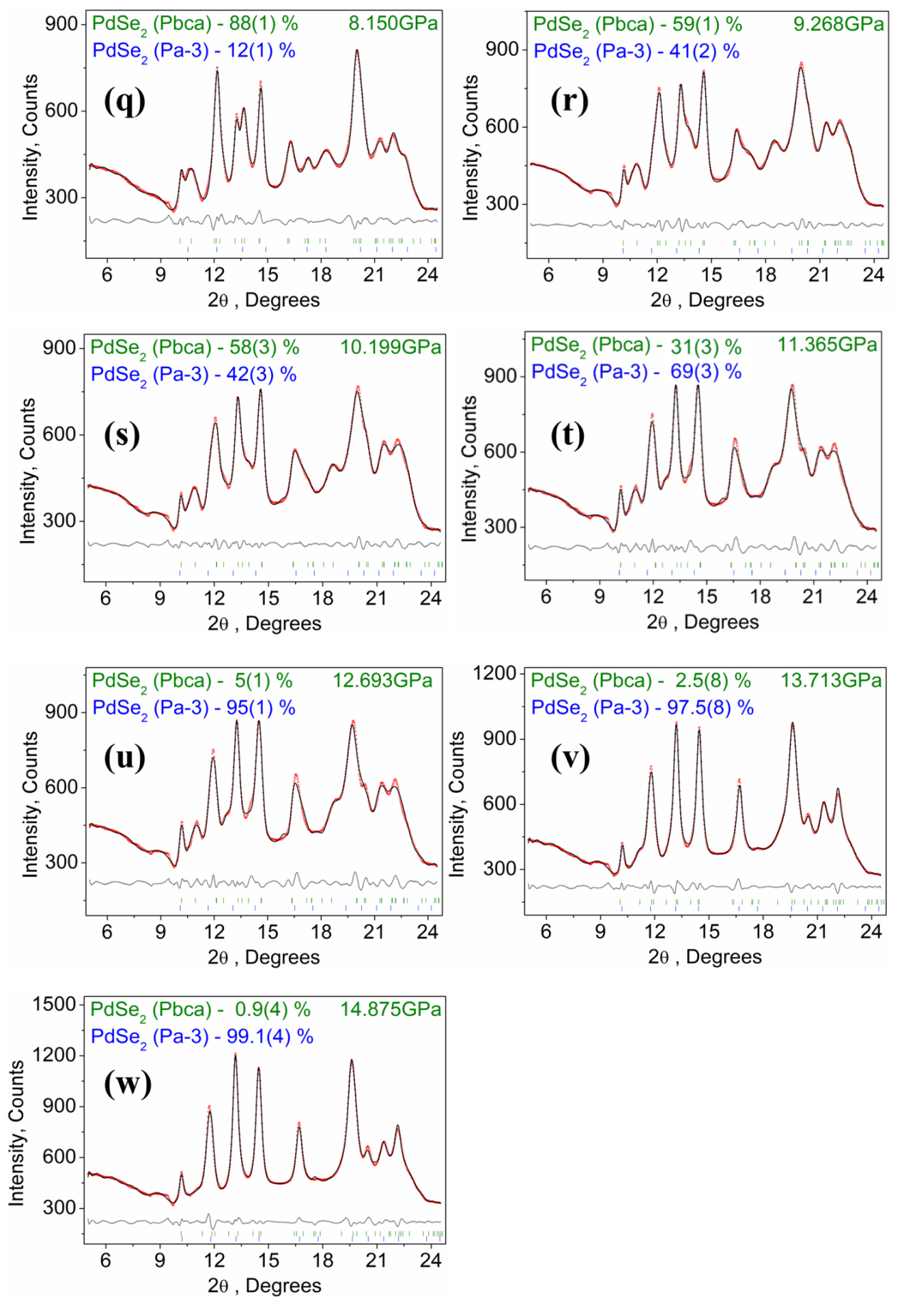
**

**Table S1** Refined cell parameters and weight of the two phases at various hydrostatic pressures

|  | **Pressure (GPa)** | ***a*(Å)** | ***b*(Å)** | ***c*(Å)** | **Weight, %** |
| --- | --- | --- | --- | --- | --- |
| **P*bca* (LP)** | **0** | 5.7377 (7) | 5.8603 (7) | 7.6846 (11) | 100 |
|  | **0.16** | 5.7309 (12) | 5.8503 (13) | 7.672 (2) | 100 |
|  | **0.53** | 5.7363 (15) | 5.855 (2) | 7.662 (2) | 100 |
|  | **0.75** | 5.7330 (16) | 5.850 (2) | 7.610 (2) | 100 |
|  | **0.93** | 5.735 (17) | 5.851 (2) | 7.606 (2) | 100 |
|  | **1.13** | 5.733 (17) | 5.847 (2) | 7.575 (2) | 100 |
|  | **1.49** | 5.7349 (12) | 5.8484 (13) | 7.531 (2) | 100 |
|  | **1.80** | 5.7345 (12) | 5.8470 (13) | 7.487 (2) | 100 |
|  | **2.16** | 5.7329 (11) | 5.8435 (13) | 7.449 (2) | 100 |
|  | **2.73** | 5.7290 (12) | 5.8401 (13) | 7.384 (2) | 100 |
|  | **3.43** | 5.7295 (14) | 5.8353 (14) | 7.316 (2) | 100 |
|  | **3.94** | 5.7312 (14) | 5.8337 (15) | 7.260 (2) | 100 |
|  | **4.67** | 5.742 (2) | 5.839 (2) | 7.165 (2) | 100 |
|  | **5.51** | 5.7555 (12) | 5.8467 (13) | 7.040 (2) | 100 |
|  | **6.21** | 5.771 (2) | 5.857 (2) | 6.982 (4) | 86 (6) |
|  | **7.21** | 5.800 (3) | 5.876 (3) | 6.845 (5) | 81 (1) |
|  | **8.15** | 5.806 (3) | 5.896 (3) | 6.660 (4) | 88 (1) |
|  | **9.27** | 6.02 (1) | 5.93 (1) | 6.600 (1) | 59 (2) |
|  | **10.20** | 6.047 (8) | 5.952 (8) | 6.594 (9) | 57 (3) |
|  | **11.37** | 6.10 (1) | 6.01 (1) | 6.54 (1) | 31 (3) |
|  | **12.69** | 6.055 (5) | 5.942 (6) | 6.460 (7) | 5 (1) |
|  | **13.71** | 6.016 (6) | 5.960 (7) | 6.370 (8) | 2.5 (8) |
|  | **14.88** | 5.999 (7) | 5.915 (7) | 6.277 (11) | 0.9 (4) |
|  | **Pressure (GPa)** | ***a*(Å)** | | | **Weight, %** |
| **P*a-3* (HP)** | **6.21** | 5.95 (1) | | | 14 (6) |
|  | **7.21** | 5.872 (4) | | | 9 (1) |
|  | **8.15** | 5.858 (5) | | | 12 (1) |
|  | **9.27** | 6.09 (3) | | | 41 (2) |
|  | **10.20** | 6.10 (1) | | | 43 (3) |
|  | **11.37** | 6.12 (5) | | | 69 (3) |
|  | **12.69** | 6.072 (4) | | | 95 (1) |
|  | **13.71** | 6.049 (4) | | | 97.5 (8) |
|  | **14.88** | 6.0271 (4) | | | 99.1 (4) |

**Figure S3** XRD patterns of PdSe_2_ at various hydrostatic pressures between 0 and 12.19 GPa with pressure-loading and –unloading process.

**
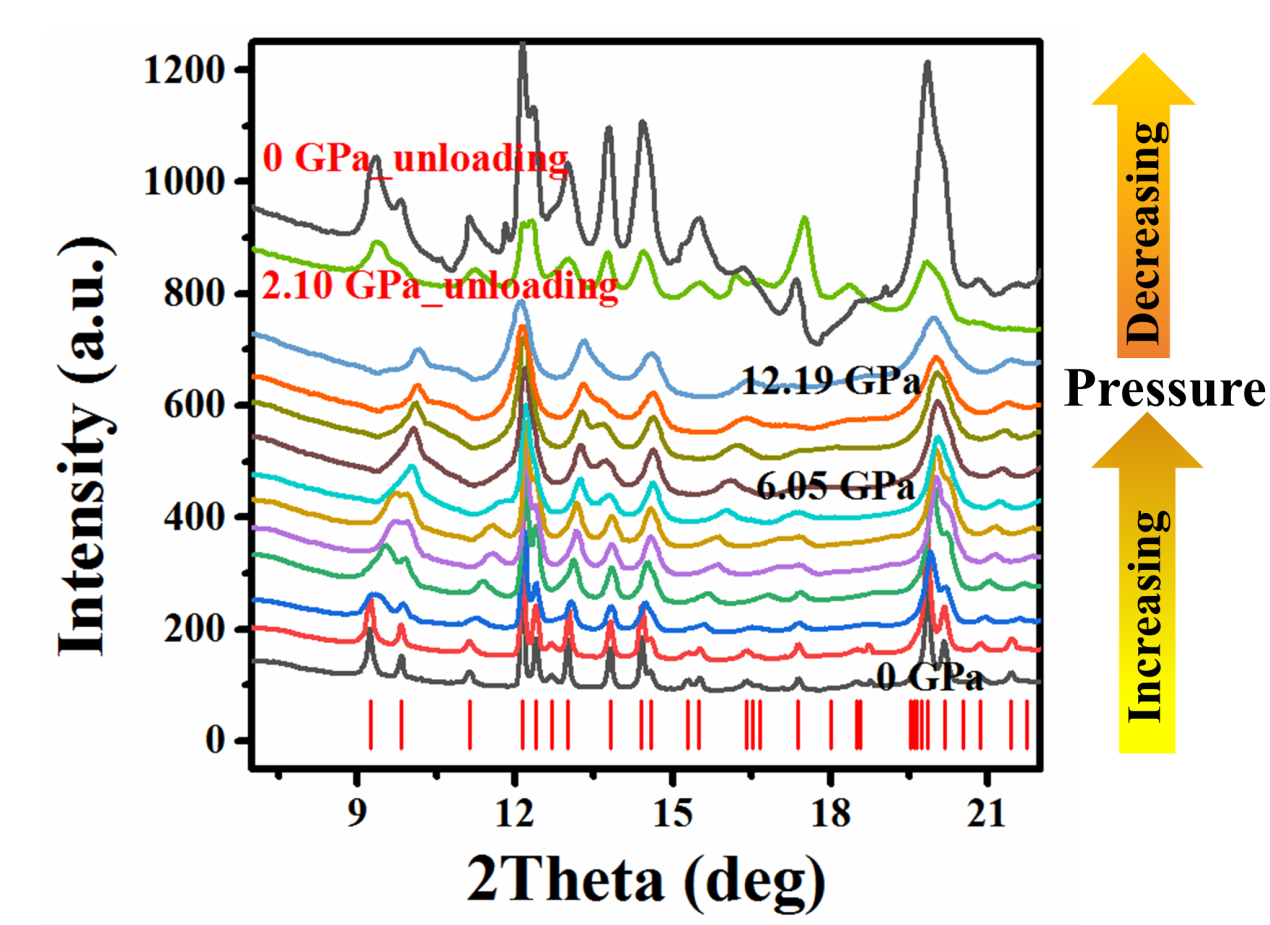
**

**Figure S4** Refinement plots of PdSe_2_ with pressure-loading and -unloading process at (a) 0 GPa, (b) 0.31 GPa, (c) 0.79 GPa, (d) 1.48 GPa, (e) 2.85 GPa, (f) 3.99 GPa, (g) 4.99 GPa, (h) 6.05 GPa, (i) 7.94 GPa, (j) 9.97 GPa, (k) 12.19 GPa, (l) 2.10 GPa for pressure unloading, (m) 0 GPa for pressure unloading. Clearly, after the pressure-releasing back to 0GPa, the Pa-3 phase disappears and only Pbca phase is left**,** demonstrating that the pressure-induced phase-transition is reversible.

**
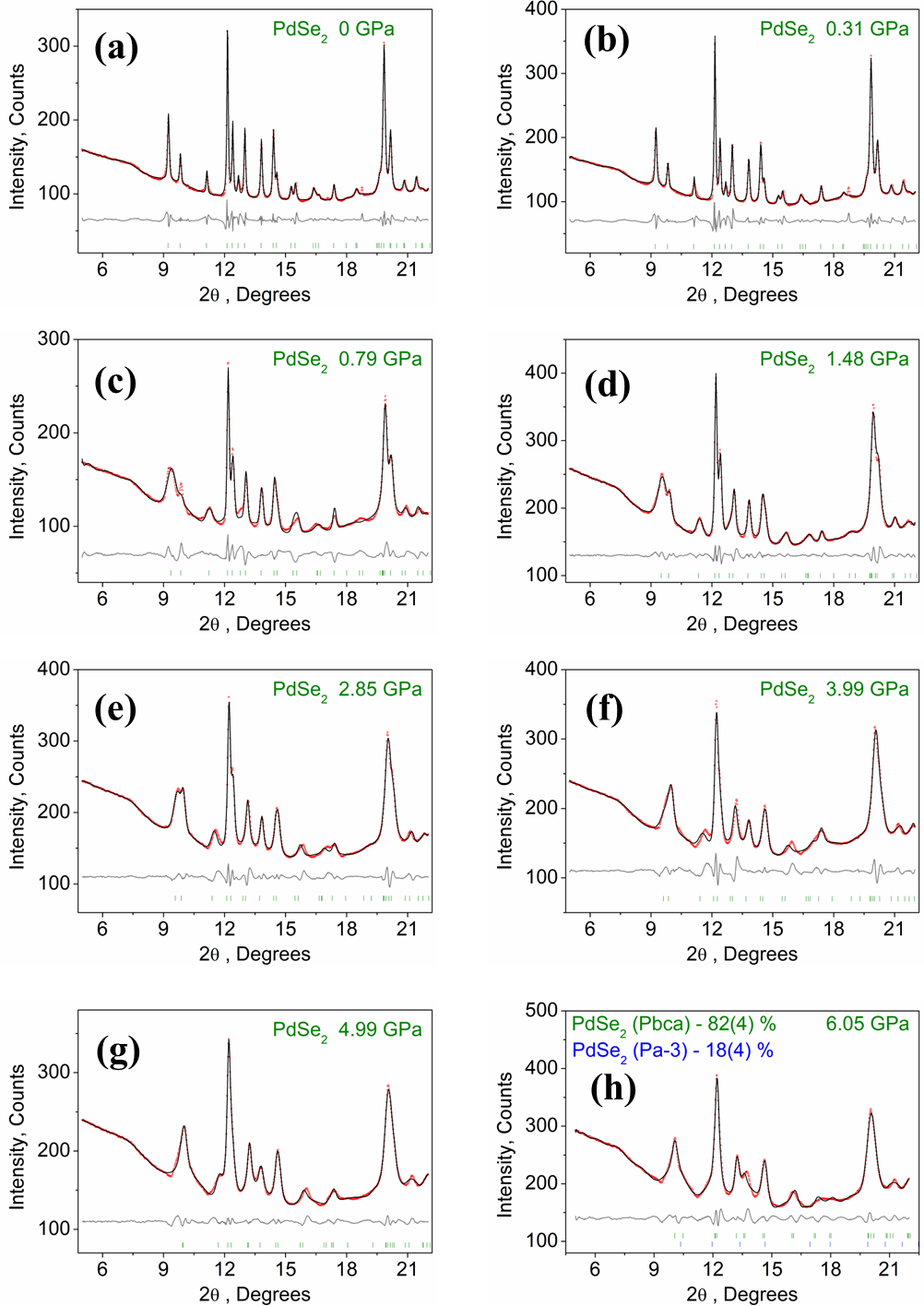
**

**
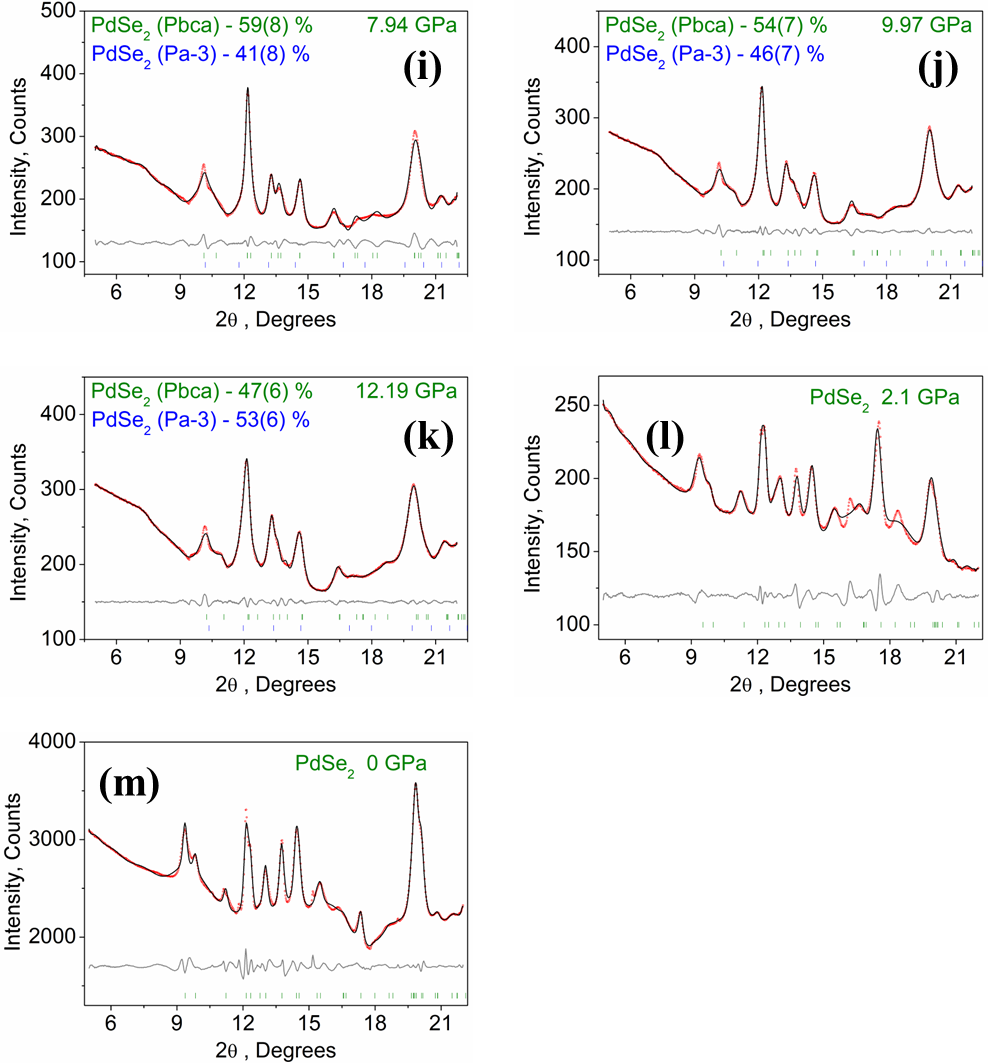
**

**Figure S5** Dependence of lattice volume on pressure. After the pressure-releasing to 0 GPa, the lattice volume almost returns back to the initial value before the pressure uploading process, which demonstrates that the variation of cell parameter in the *Pbca* phase *versus* pressure is reversible.





**Table S2** Area compressibility (AC) and NAC pressure range of all known NAC materials

|  | Materials | AC (/TPa) | NAC Pressure range (GPa) | ΔP (GPa) | Ref. |
| --- | --- | --- | --- | --- | --- |
| Lifshitz mechanism | PdSe_2_ | -13.14(239) | 3.94 -11.37 | 7.43 | This work |
|  | KBBF *R*32 | -1.0(2) | 0.22-6.39 | 6.17 | (1) |
|  | KBBF *R*-3c | -1 | 3-10 | 7 | (2) |
|  | NaV_2_O_5_ | -1.5 | 4-10 | 6 | (3) |
|  | Ag(tcm) | -7.5(8) | 0-0.62 | 0.62 | (4) |
|  | TlGaSe_2_  Zn(CH_3_COO)_2_.2H_2_O | -4.99  -8.1(8) | --  0.15-4.44 | --  4.29 | (5)  (6) |
| Other mechanisms | 2MeBzIm | -15(6) | 0.24-2.40 | 2.16 | (7) |
|  | [Zn(L)_2_(OH)_2_]_n_.guest | -72(6) | 1.0-2.6 | 1.6 | (8) |
|  | Oxalic acid dehydrate | -36.71 | 0-0.85 | 0.85 | (9) |
|  | CrAs | -2.11 | 0-0.6 | 0.6 | (10) |

**Table S3.** Bond length and angles evolution *versus* pressure.

| **Pressure (GPa)** | **Intralayer Pd-Se1(Å)** | **Intralayer Pd-Se2(Å)** | **Interlayer**  **Pd-Se(Å)** | **∠Pd-Se-Pd(°)** |
| --- | --- | --- | --- | --- |
| 0.00 | 2.469 | 2.477 | 3.209 | 111.858 |
| 0.16 | 2.467 | 2.478 | 3.198 | 111.791 |
| 0.53 | 2.471 | 2.478 | 3.194 | 111.819 |
| 0.75 | 2.471 | 2.477 | 3.166 | 111.707 |
| 0.93 | 2.471 | 2.478 | 3.165 | 111.748 |
| 1.13 | 2.471 | 2.477 | 3.149 | 111.673 |
| 1.49 | 2.473 | 2.478 | 3.127 | 111.658 |
| 1.80 | 2.474 | 2.476 | 3.106 | 111.646 |
| 2.16 | 2.471 | 2.477 | 3.087 | 111.598 |
| 2.73 | 2.471 | 2.477 | 3.054 | 111.518 |
| 3.43 | 2.471 | 2.477 | 3.021 | 111.472 |
| 3.94 | 2.471 | 2.477 | 2.994 | 111.463 |
| 4.67 | 2.475 | 2.477 | 2.952 | 111.576 |
| 5.51 | 2.477 | 2.482 | 2.895 | 111.639 |
| 6.21 | 2.481 | 2.485 | 2.872 | 111.780 |
| 7.21 | 2.487 | 2.490 | 2.816 | 112.072 |
| 8.15 | 2.491 | 2.493 | 2.733 | 112.221 |
| 9.27 | 2.513 | 2.548 | 2.738 | 113.209 |
| 10.20 | 2.520 | 2.557 | 2.740 | 113.364 |
| 11.37 | 2.540 | 2.574 | 2.727 | 113.705 |
| 12.69 | 2.516 | 2.556 | 2.680 | 113.475 |
| 13.71 | 2.517 | 2.543 | 2.641 | 113.622 |
| 14.88 | 2.501 | 2.530 | 2.599 | 113.699 |

**Figure S6** High-pressure Raman spectra of PdSe_2_ from 0.6 GPa to 12.3 GPa. The small peaks on the left side are due to the tiny amorphous impurity which cannot be detected in XRD data, or the background noise from the instrument. The scattering signal of the amorphous impurity is immersed in the background in the XRD data, and does not interfere the accurate determination of crystal structure.


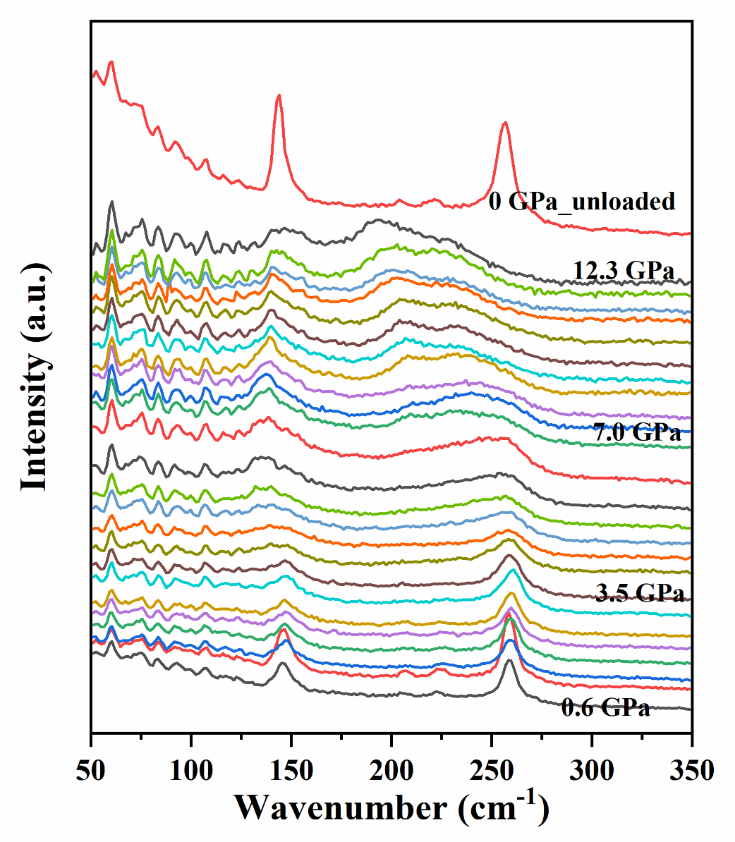


**Figure S7** Raman spectrum and the atomic vibration assignment of the Raman peaks. (a) and (b) for low-pressure phase at ambient pressure; (c) and (d) for high-pressure phase at 12.3GPa.


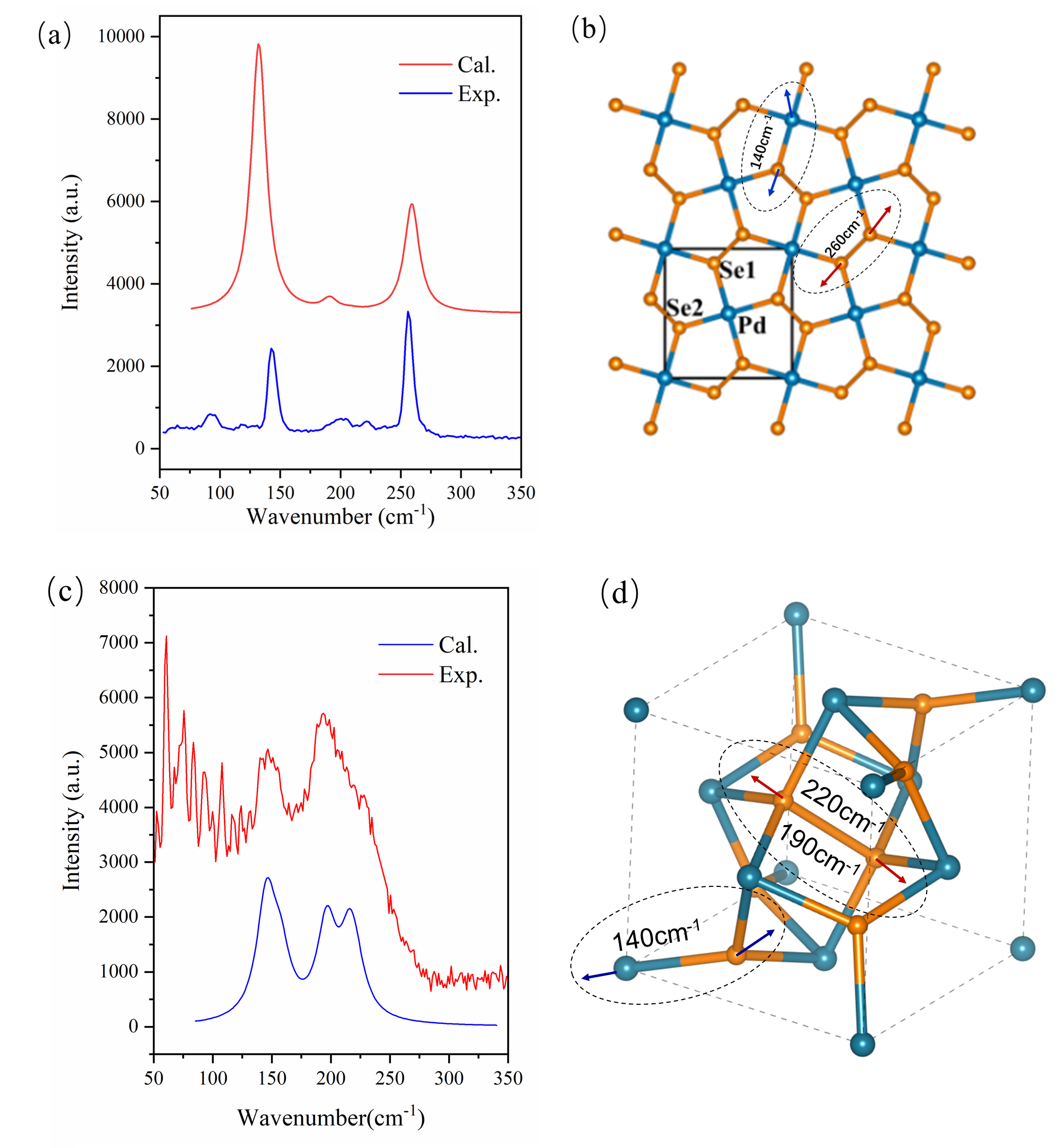


**Figure S8** Charge difference density contours of PdSe_2_ at various hydrostatic pressures from 0 to 14.88 GPa (totally 23 pressures). Palladium and selenium are represented by peacock blue and orange balls, respectively. The blue and red solid-line ellipses indicate the charge-gain and charge-loss areas along the intralayer Pd-Se bonds, while the dashed-line ones indicate the charge-gain and charge-loss areas along the interlayer Pd-Se pair, respectively. The size and shape for each ellipse are set to be kept constant with respect to pressure in order to make the comparison of charge-density difference explicit. The charge difference density contours during the NAC range are highlight by red boxes.

**
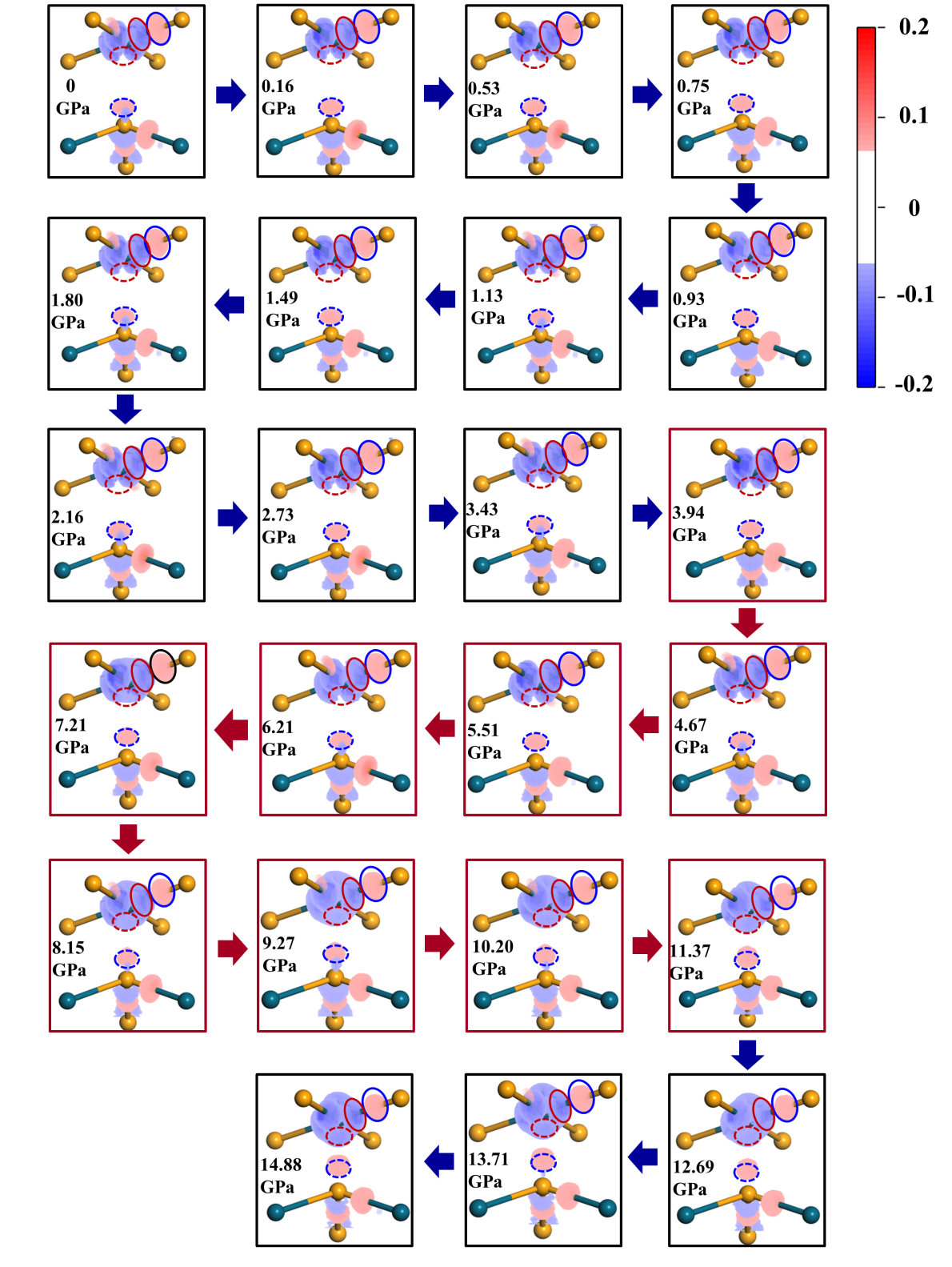
**

**Figure S9** Charge difference density contours of PdSe_2_ at some specified pressures. The magnified charge-difference densities on the [PdSe_2_] layer and on the neighboring Se atom in the NAC pressure range (3.94GPa, 6.21GPa, 9.27GPa and 11.37GPa) are displayed on left and right corners, respectively.

**
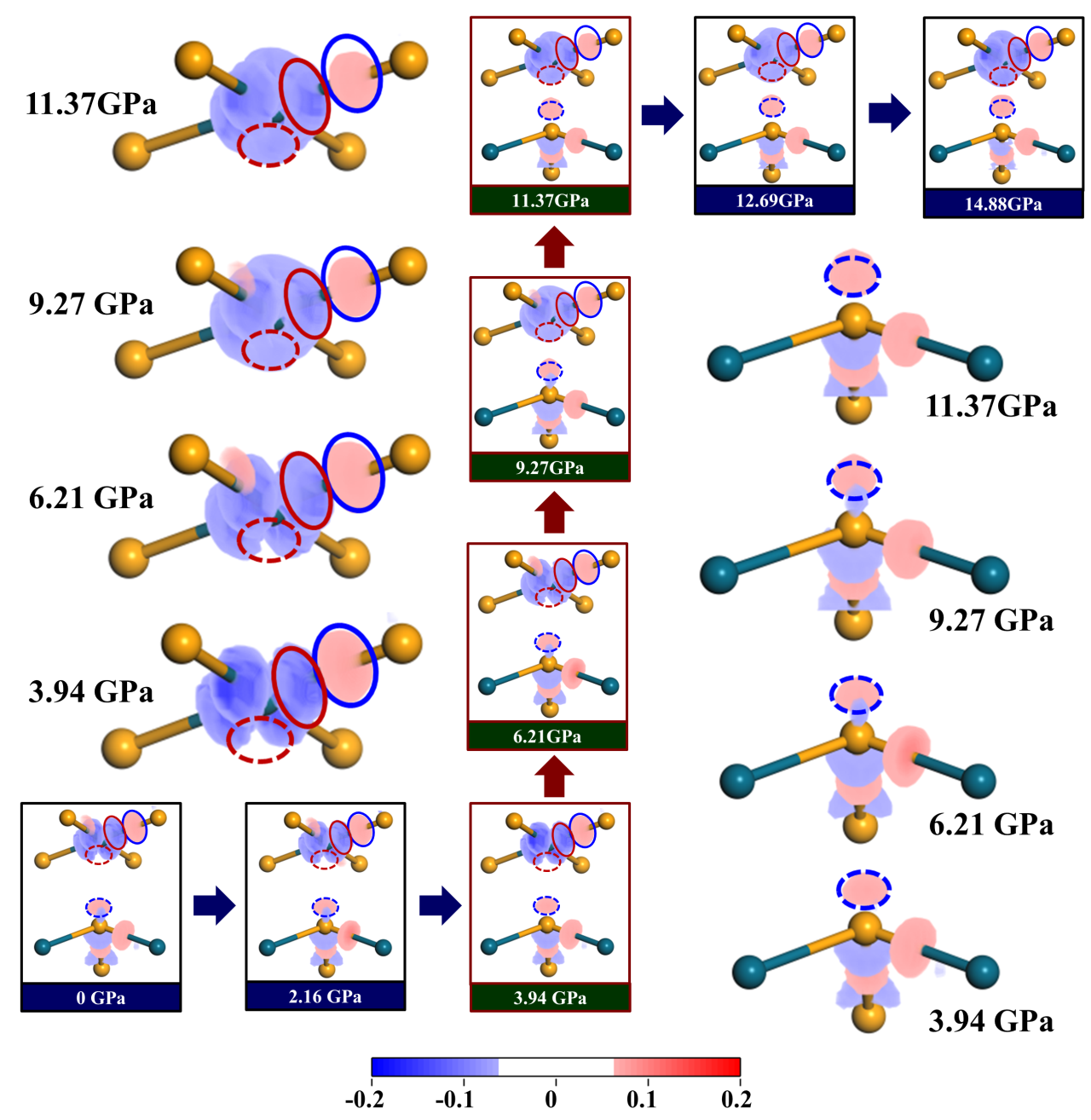
**

**Figure S10** Photos of PdSe_2_ sample: (a) the as-synthesized powder samples and (b) samples in the diamond anvil cell. According to the X-ray diffraction spectrum @ 0GPa plotted in Figure S2a, all the peaks can be indexed to PdSe_2_ and no impurity peak is detected, which verifies the high purity of the sample. Moreover, all the peaks are sharp, with the full widths at half maximum (FWHM) less than ~0.1 degree, and this demonstrates the high crystallinity of the sample. Additionally, the sharp peaks (with the FWHM less than ~10cm^-1^) are also manifested in Raman spectrum @0GPa (blue line in Figure S7a), which also confirm the high crystallinity. Therefore, the sample is of a high quality to guarantee the accuracy of the crystal structures determined from the X-ray data and thus the conclusion of this work.


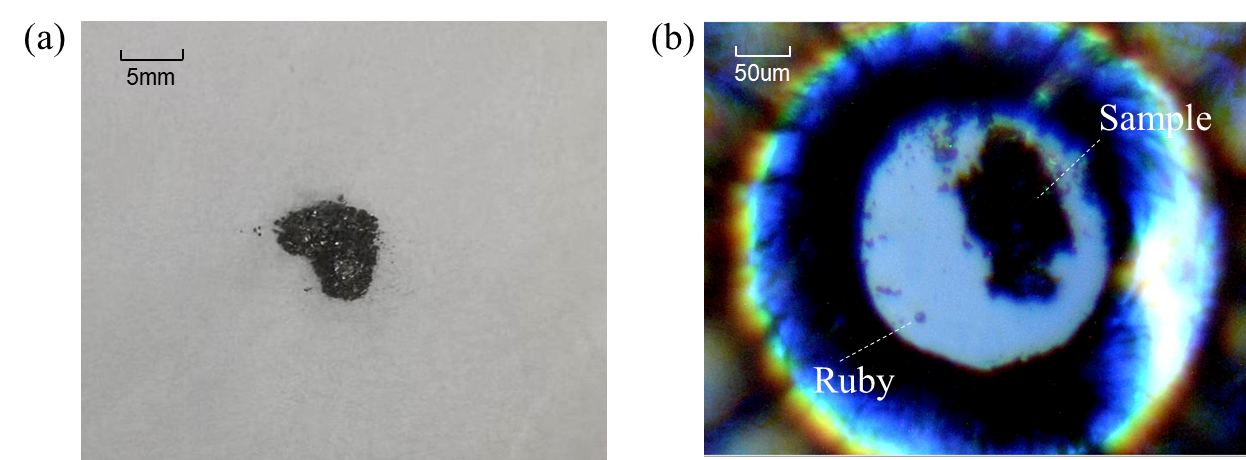


**Figure S11** Schematic for the setup of high pressure and X-ray


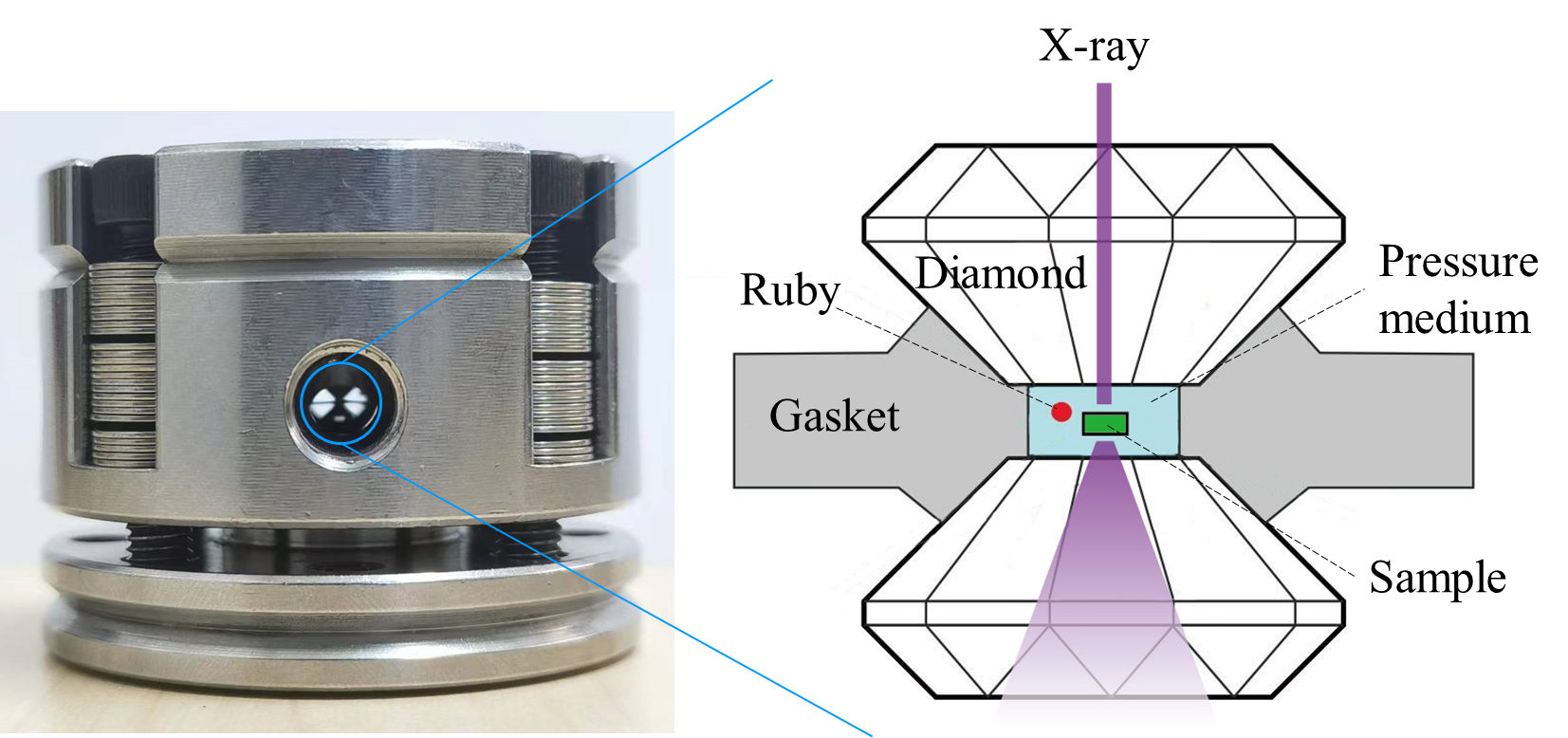


**Section S1** Discussion on the situation that the determination of NAC is not affected by the phase transition, and the explanation about the abrupt change in lattice parameter between 8.15 and 9.27GPa .

It is known that the determination of cell parameters with respect to pressure is crucial to investigate the compressibility behavior in a material (see, *e.g.*, zinc dicyanoaurate (11)). For the low-pressure phase in PdSe_2_ (with an orthogonal space group Pbca) there are three independent cell parameters (a, b, and c), while for the high-pressure phase (with a cubic space group Pa-3) there is only one independent cell parameter (a = b = c). So there are totally four cell parameters that influence the XRD peak positions. In comparison, there are 49 XRD peaks used for structural refinement. The ratio (49:4=~12) of peak number to refined parameters is high enough for the cell parameter determination. Meanwhile, owing to a lower symmetry for the orthogonal space group Pbca, there exist at least ten XRD peaks solely belonging to the low-pressure phase, which is also sufficient to refine its (three) cell parameters. Our study revealed that the errors of the refined cell parameters in PdSe_2_ are very small and located in the range of 0.01% ~ 0.17%, and the values of cell parameters within 2-3 esd are trustworthy. These values are much smaller than the change of *a*- and *b*-axis (6.4% and 3.0%, respectively) in the NAC range. This means that the determination of the NAC magnitude in PdSe_2_ was not disturbed by the coexistence of two phases.

The abrupt change of the lattice parameters between approximately 8 and 11GPa (8.15 GPa and 9.27 GPa) is owing to the strong correlation of the refined cell parameters between the low-pressure and high-pressure phases, especially when their weight ratio is approached. According to the mathematic method to deal with the correlation matrix in the refinement, the correlation between the crystal structures of the constituent phases reaches the maximum in the multi-phase XRD data as the weight ratio of the two phases is equal. The phase weight between low-pressure and high-pressure phases is sharply changed from 88:12 to 59:41 with the pressure increased from 8.15 to 9.27 GPa, so the correlation between the refined parameters of these two phases is sharply enhanced and the lattice parameters of the low-pressure phase is abruptly increased. For the pressures far away from 8.15 GPa and 9.27 GPa, the lattice parameters show a gradual and smooth change.

**Reference**

1 Jiang, X, Luo, S, Kang, L*, et al.* Isotropic Negative area compressibility over large pressure range in potassium beryllium fluoroborate and its potential applications in deep ultraviolet region. *Adv. Mater.* 2015; **27**(33): 4851-7.

2 Yu, D H, Avdeev, M, Sun, D H*, et al.* Understanding the unusual response to high pressure in KBe_2_BO_3_F_2_. *Sci Rep*. 2017; **7**(1): 4027.

3 Loa, I, Syassen, K, Kremer, R K*, et al.* Structural properties of NaV_2_O_5_ under high pressure. *Phys. Rev. B* 1999; **60**(10): R6945-R8.

4 Hodgson, S A, Adamson, J, Hunt, S J*, et al.* Negative area compressibility in silver(I) tricyanomethanide. *Chem. Commun.* 2014; **50**(40): 5264-6.

5 Seyidov, M Y, Suleymanov, R A. Negative thermal expansion due to negative area compressibility in TlGaSe_2_ semiconductor with layered crystalline structure. *J. Appl. Phys.* 2010; **108**(6): 063540.

6 Feng, G, Zhang, W-X, Dong, L*, et al.* Negative area compressibility of a hydrogen-bonded two-dimensional material. *Chem. Sci.* 2019; **10**(5): 1309-15.

7 Zieliński, W, Katrusiak, A. Colossal monotonic response to hydrostatic pressure in molecular crystal induced by a chemical modification. *Cryst. Growth Des.*2014; **14**(9): 4247-53.

8 Cai, W, Gladysiak, A, Aniola, M*, et al.* Giant negative area compressibility tunable in a soft porous framework material. *J. Am. Chem. Soc.* 2015; **137**(29): 9296-301.

9 Colmenero, F, Jiang, X, Li, X*, et al.* Negative area compressibility in silver oxalate. *J. Mater. Sci.* 2021; **56**(1): 269-77.

10 Yu, Z, Wu, W, Hu, Q*, et al.* Anomalous anisotropic compression behavior of superconducting CrAs under high pressure. *Roc. Natl. Acad. Sci. U. S. A.* 2015; **112**(48): 14766-70.

11 B, Andrew C, Jadna, C, Claire, L, *et al*. Giant negative linear compressibility in zinc dicyanoaurate. *Nat. Mater.* 2013; **12**(3):212-216
